# Adverse effects of escitalopram and vortioxetine in young patients with depression: a retrospective comparative study

**DOI:** 10.1186/s40780-026-00613-y

**Published:** 2026-07-25

**Authors:** Ayae Nomura, Kazuki Takayama, Ayane Miyazawa, Yuuma Higashi, Hiroaki Yoshinari, Naoki Yamamoto, Shinya Takasaki

**Affiliations:** 1 School of Pharmacy, International University of Health and Welfare, 2600-1 Kitakanemaru, Otawara, Tochigi 324-8501 Japan; 2 Department of Pharmacy, International University of Health and Welfare Hospital, 537-3 Iguchi, Nasushiobara, 329-2763 Tochigi Japan

**Keywords:** Depression in young patients, Vortioxetine, Escitalopram, Nausea, gastrointestinal medicine

## Abstract

**Background:**

Depression is a serious mental disorder that impairs both psychological and physical functioning. When depression occurs in individuals in their 20s and 30s, it may adversely affect not only the continuation of academic or occupational activities but also interpersonal relationships in educational and workplace settings. Therefore, appropriate pharmacotherapy is essential for this patient population. Although several studies have evaluated newer antidepressants in recent years, detailed information regarding adverse effects in routine clinical practice remains limited, and studies focusing specifically on younger populations are scarce. The aim of this study was to compare the incidence and characteristics of adverse events associated with two newer antidepressants frequently prescribed to young patients in clinical practice, escitalopram (Lexapro^®^) and vortioxetine (Trintellix^®^), and to explore strategies for ensuring safe pharmacotherapy.

**Methods:**

This retrospective study analyzed patients with depression who were prescribed escitalopram or vortioxetine between 2019 and 2024. Clinical data were collected from medical records, with a follow-up period of 3 months. Statistical analysis was performed using EZR, with a significance level set at *p* < 0.05.

**Results:**

The incidence of adverse events was significantly higher in the vortioxetine group than in the escitalopram group. Among the observed adverse events, nausea occurred significantly more frequently in patients receiving vortioxetine. Furthermore, a lower incidence of nausea was observed among patients who received concomitant gastrointestinal medications.

**Conclusions:**

The findings of this study suggest that concomitant gastrointestinal medication use may be associated with a lower incidence of nausea among young patients treated with vortioxetine. Although causal relationships cannot be established because of the retrospective study design, these findings may provide preliminary real-world evidence regarding the management of vortioxetine-associated nausea. Further prospective studies are needed to confirm these observations.

## Background

Depression is a major global health concern that significantly affects not only patients but also their families and society [1]. In young adults, depression is associated with serious outcomes, including substance abuse, social dysfunction, self-harm, and suicide, and is characterized by a high risk of recurrence [2, 3]. Therefore, establishing pharmacological treatment strategies that ensure both safety and tolerability is particularly important in this population.

Escitalopram, a selective serotonin reuptake inhibitor (SSRI), is one of the most widely prescribed antidepressants, with well-established efficacy and safety [4, 5]. Vortioxetine, which acts as both a serotonin reuptake inhibitor and a serotonin receptor modulator, has a novel multimodal mechanism of action and has been increasingly prescribed in clinical practice in recent years [6–8]. Although several studies have reported the efficacy and safety of these antidepressants [9, 10], real-world comparative data on the incidence and severity of adverse effects remain limited, particularly in young patients with depression [11].

Adverse effects are important factors that can lead to poor medication adherence and treatment discontinuation, thereby affecting clinical outcomes [12]. In particular, gastrointestinal symptoms such as nausea and vomiting are relatively common with antidepressants and are known to significantly affect tolerability [13]. In Japan, suicide is one of the leading causes of death among young people and is closely associated with depression, highlighting the clinical importance of optimizing antidepressant tolerability in this age group [14].

Therefore, this study aimed to compare the incidence of adverse effects between escitalopram and vortioxetine in adults aged 20–39 years and to examine approaches to minimizing adverse effects and ensuring safe pharmacotherapy.

## Methods

### Study design and data collection

This retrospective study identified outpatients diagnosed with depression at the Department of Psychiatry of the International University of Health and Welfare Hospital and the International University of Health and Welfare Clinic who were prescribed escitalopram or vortioxetine between April 1, 2019, and March 31, 2024. Young patients were defined as adults aged 20–39 years.

Patient information was collected from electronic and paper medical records at each facility, as well as through confirmation with psychiatrists. The medical records included basic patient information, physician examination records, test results, medication therapy and administration records, and adverse event records. Patients diagnosed with depression were identified based on the International Classification of Diseases, 10th Revision (ICD-10).

The observation period was defined as 3 months after the initiation of escitalopram or vortioxetine. This period was selected because adverse events associated with antidepressant initiation, particularly gastrointestinal symptoms such as nausea, are most commonly observed during the early phase of treatment and may influence treatment continuation. Adverse events occurring during this period were included in the analysis. Adverse events were identified from physician-documented medical records and patient-reported symptoms recorded during routine clinical practice. All medical records were generated by treating psychiatrists as part of routine clinical care. Adverse-event information was extracted from outpatient consultation records, medication management records, and adverse-event documentation available in the medical charts. No standardized adverse-event assessment scale or structured questionnaire was routinely used during the study period.

Eligible patients were identified from outpatient psychiatric records; therefore, inpatients were not included in the initial patient identification process. Patients who were transferred to another institution and could not be followed, those with restricted access to medical records, and those with unclear clinical outcomes were excluded from the analysis. At both institutions, escitalopram and vortioxetine prescribed to eligible patients were standardized to the branded formulations, Lexapro^®^ 10 mg and Trintellix^®^ 10 mg, for analysis. The initial doses of escitalopram and vortioxetine were 5 mg or 10 mg once daily. The dose of each antidepressant was adjusted within a range of 5–20 mg/day at the discretion of the treating psychiatrist. However, most patients in the present study received 10 mg/day for both medications.

This study was approved by the Ethics Review Committee of the International University of Health and Welfare (approval number: 24-Io-12-2).

### Statistical analysis

Continuous variables are presented as medians with interquartile ranges (IQRs) and were compared between groups using the Mann–Whitney U test. Categorical variables are presented as numbers and percentages and were compared using Fisher’s exact test.

To evaluate the association between antidepressant type and the occurrence of adverse effects while adjusting for potential confounders, logistic regression analysis was performed with the presence of adverse effects as the dependent variable. Age and sex were included as covariates in the multivariable model, selected a priori based on their clinical relevance. Odds ratios (ORs) with 95% confidence intervals (CIs) were calculated.

The incidence of individual adverse effects was compared between the escitalopram and vortioxetine groups using Fisher’s exact test. In addition, among patients treated with vortioxetine, the incidence of nausea was compared according to the use of concomitant gastrointestinal medications using Fisher’s exact test.

All statistical analyses were performed using EZR (Saitama Medical Center, Jichi Medical University, Saitama, Japan), a graphical user interface for R (The R Foundation for Statistical Computing, Vienna, Austria) [15]. A two-sided *p* value < 0.05 was considered statistically significant.

## Results

### Patient characteristics

Baseline characteristics of the patients are shown in Table 1. The median age was significantly higher in the escitalopram group than in the vortioxetine group (29 [IQR 25–34] vs. 24 [IQR 22–29], *p* = 0.004). There were no significant differences in sex, initial dose, or treatment status between the two groups.

**Table 1:** Baseline characteristics of patients treated with escitalopram or vortioxetine

	Escitalopram (n = 53)	Vortioxetine (n = 48)	p value
Age, median (IQR)	29 (25–34)	24 (22–29)	0.004
Sex, n (%)			1
Male	14 (26.4)	13 (27.1)	
Female	39 (73.6)	35 (72.9)	
Initial dose, n (%)			0.822
5 mg	13 (24.5)	13 (27.1)	
10 mg	40 (75.5)	35 (72.9)	
Treatment status, n (%)			0.45
First antidepressant treatment	39 (73.6)	32 (66.7)	
Switched from another antidepressant	14 (26.4)	16 (33.3)	

Values are presented as median (interquartile range) or number (%). Continuous variables were compared using the Mann–Whitney U test, and categorical variables were compared using Fisher’s exact test

### Factors associated with adverse events

The results of the logistic regression analysis are shown in Table 2. In the unadjusted analysis, treatment with vortioxetine was associated with significantly higher odds of adverse events compared with escitalopram (odds ratio [OR] 3.24, 95% confidence interval [CI] 1.43–7.35, *p* = 0.005). This association remained statistically significant after adjustment for age and sex (OR 3.25, 95% CI 1.39–7.63, *p* = 0.007). Age, sex, and starting dose were not significantly associated with the occurrence of adverse events.

**Table 2:** Logistic regression analysis of factors associated with adverse events

Variable	Unadjusted OR(95% CI)	pvalue	Adjusted OR*(95% CI)	pvalue
Antidepressant (vortioxetine vs escitalopram)	3.24 (1.43–7.35)	0.005	3.25 (1.39–7.63)	0.007
Age(per 1-year increase)	0.97 (0.91–1.05)	0.474	0.99 (0.92–1.08)	0.875
Sex (male vs female)	1.17 (0.48–2.86)	0.730	1.27 (0.49–3.30)	0.618
Starting dose (10 mg vs 5 mg)	1.33 (0.53–3.30)	0.543	1.51 (0.56–4.10)	0.421

*Adjusted for age and sex. OR, odds ratio; CI, confidence interval

Odds ratios (ORs) with 95% confidence intervals (CIs) were estimated using logistic regression models. Both unadjusted and multivariable models are presented. The multivariable model included age and sex as covariates

### Adverse events and their management

**Fig. 1:**
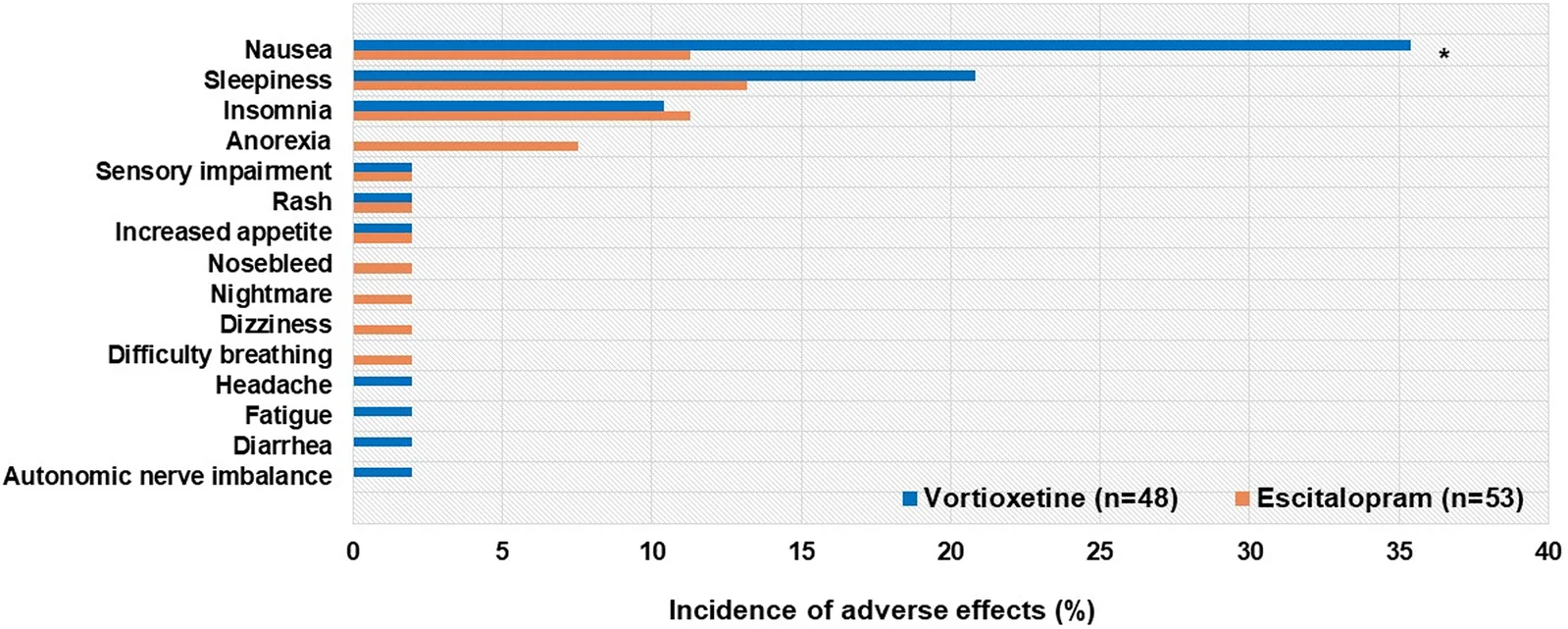
Incidence of adverse events in patients treated with escitalopram or vortioxetine. The incidence of adverse events was compared between the escitalopram group (*n* = 53) and the vortioxetine group (*n* = 48). The incidence of nausea was significantly higher in the vortioxetine group than in the escitalopram group (35.4% vs. 11.3%, *p* = 0.005). Comparisons between groups were performed using Fisher’s exact test. * indicates a significant difference between vortioxetine and escitalopram (*p* < 0.05, Fisher’s exact test)

The incidence of adverse events by category is shown in Fig. 1, where nausea was the most frequently observed adverse effect and occurred significantly more often in the vortioxetine group than in the escitalopram group (35.4% vs. 11.3%, *p* = 0.005), whereas no statistically significant differences were observed for the other adverse events. The incidence of nausea according to concomitant gastrointestinal medication use among patients treated with vortioxetine is shown in Table 3. Nausea occurred in 6 of 28 patients (21.4%) receiving concomitant gastrointestinal medications and in 11 of 20 patients (55.0%) not receiving gastrointestinal medications (*p* = 0.0308).

**Table 3:** Association between concomitant gastrointestinal medication use and nausea in patients treated with vortioxetine

	gastrointestinal medication (+) (*n* = 28)	gastrointestinal medication (-) (*n* = 20)	*p*-value
Nausea (+)	6 (21.4)	11 (55.0)	0.0308
Nausea (-)	22 (78.6)	9 (45.0)	

Values are presented as number (%). Percentages were calculated within each gastrointestinal medication group. Comparisons between groups were performed using Fisher’s exact test

**Fig. 2:**
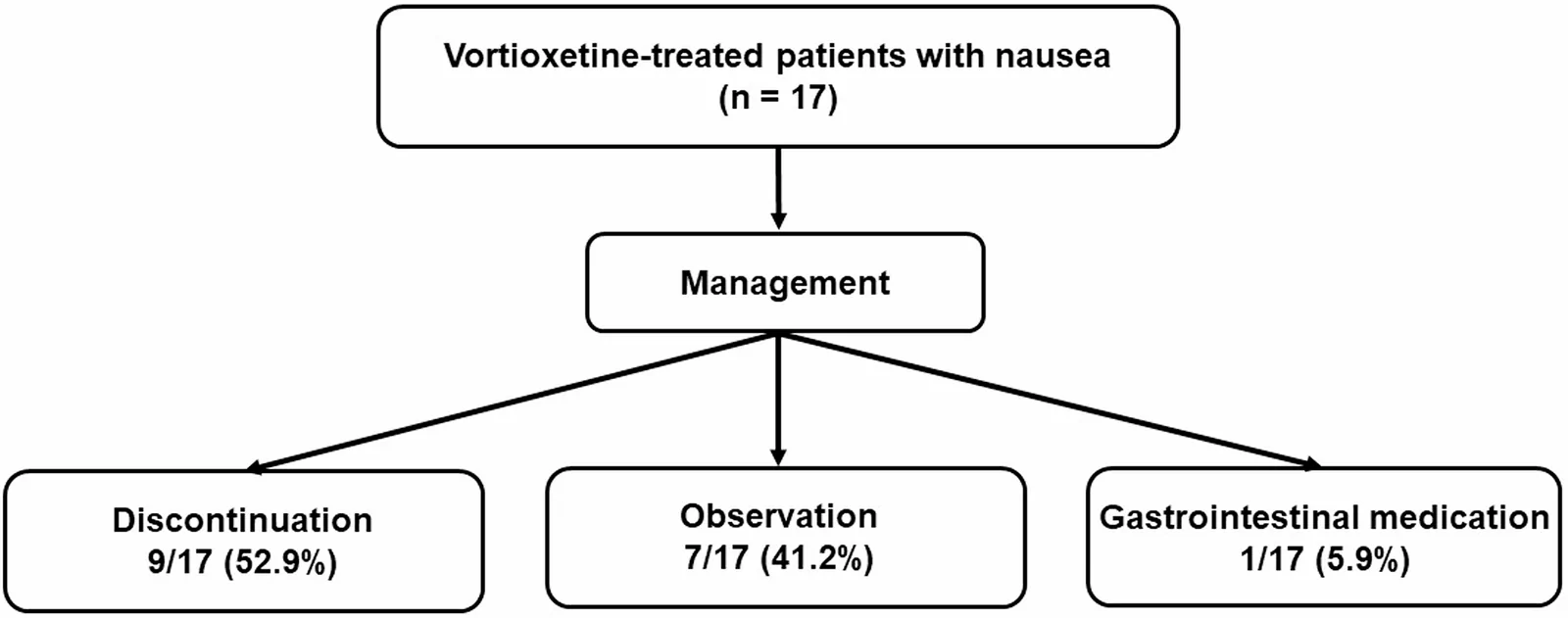
Management of nausea after its onset in patients treated with vortioxetine. Among patients who experienced nausea during vortioxetine treatment (*n* = 17), treatment was discontinued in 9 of 17 patients (52.9%), continued with observation in 7 of 17 patients (41.2%), and gastrointestinal medication was initiated after nausea onset in 1 of 17 patients (5.9%)

**Fig. 3:**
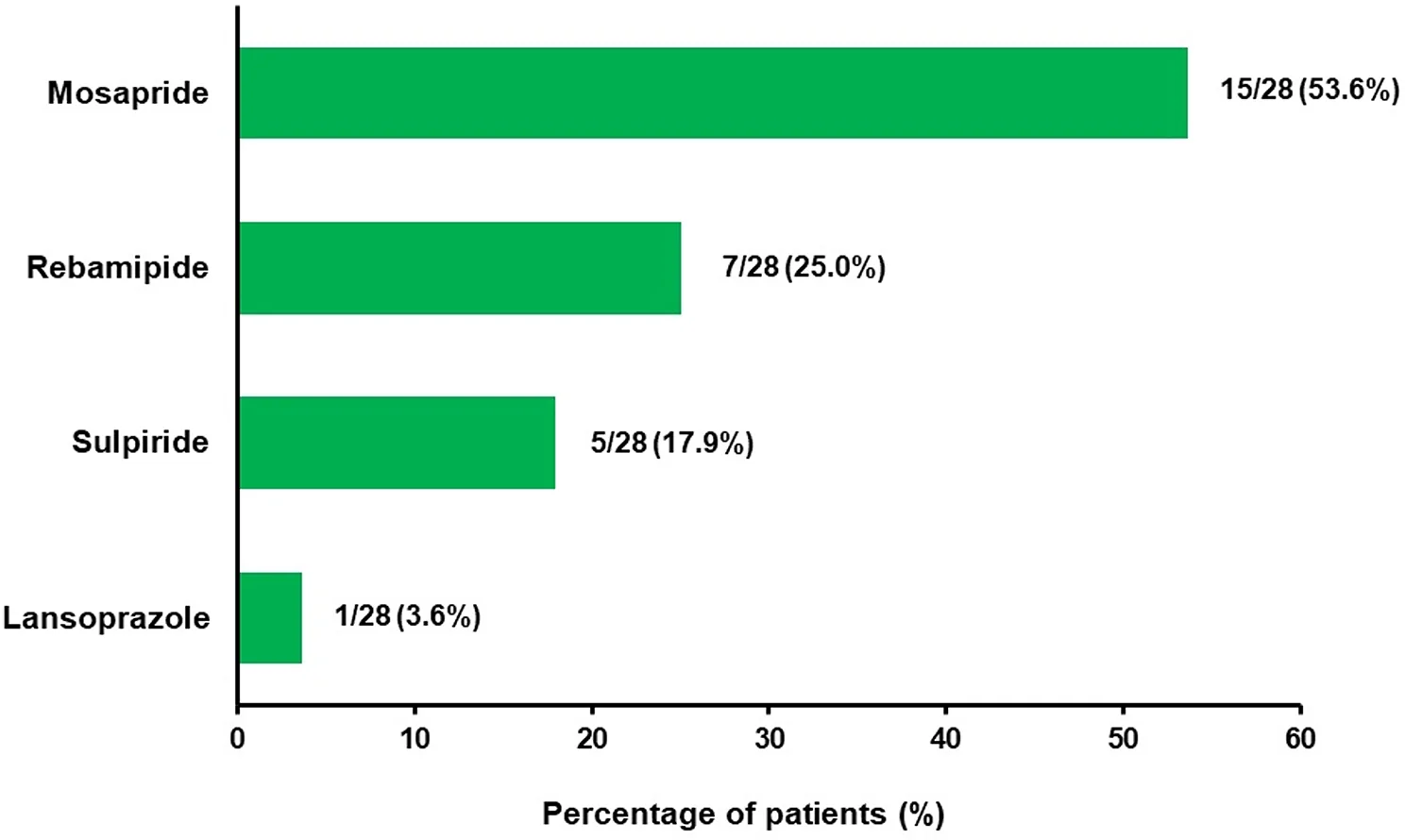
Concomitant use of gastrointestinal medications at the initiation of antidepressant treatment. Among patients who received concomitant gastrointestinal medications at antidepressant initiation (*n* = 28), mosapride was the most frequently prescribed agent in 15 of 28 patients (53.6%), followed by rebamipide in 7 of 28 patients (25.0%), sulpiride in 5 of 28 patients (17.9%), and lansoprazole in 1 of 28 patients (3.6%)

In patients treated with vortioxetine who developed nausea, management strategies are illustrated in Fig. 2. Discontinuation was the most common approach, occurring in 9 of 17 patients (52.9%), followed by continued treatment with observation in 7 of 17 patients (41.2%) and initiation of gastrointestinal medication after nausea onset in 1 of 17 patients (5.9%). Additionally, the concomitant use of gastrointestinal medications at the initiation of antidepressant treatment is presented in Fig. 3. This analysis included all patients who received gastrointestinal medications concomitantly from the start of antidepressant therapy, regardless of whether nausea subsequently occurred. Mosapride was the most frequently used agent in 15 of 28 patients (53.6%), followed by rebamipide in 7 of 28 patients (25.0%), sulpiride in 5 of 28 patients (17.9%), and lansoprazole in 1 of 28 patients (3.6%).

## Discussion

In this study, the incidence of adverse events was higher in the vortioxetine group than in the escitalopram group, with nausea occurring at a significantly higher rate. Previous studies in adult patients with depression have also reported a higher incidence of nausea with vortioxetine compared with escitalopram [11, 16]. This finding is consistent with the approved prescribing information for vortioxetine, in which nausea is listed among the most frequently reported adverse reactions [17, 18]. In a Phase III trial of Japanese patients aged 20–75 years with major depressive disorder, administration of 10 mg vortioxetine for 8 weeks resulted in adverse events in 76.2% of patients, with nausea being the most frequent (28.7%) [19]. These findings are consistent with the trends observed in the present study. Regarding adverse events other than nausea, no significant differences were observed between the escitalopram and vortioxetine groups. Previous studies in adults have also reported no significant differences in the incidence of somnolence or insomnia, and similar trends were observed in the younger patients in this study [20]. In contrast, decreased appetite was observed only in the escitalopram group. Previous research has reported that tolerance to gastrointestinal disturbances is lower in patients treated with escitalopram compared with vortioxetine [21]. Moreover, adolescents and young adults are more prone to changes in weight and appetite than adults [22]. However, because the number of cases was small, this finding should be interpreted cautiously. Young patients are also more vulnerable to disruptions in academic and social activities, and even minor adverse effects may lead to medication discontinuation or self-adjustment [23]. This highlights the need for careful management of pharmacotherapy in young patients.

The results of this study indicated that nausea associated with vortioxetine is transient, with approximately 40% of patients continuing treatment. Previous studies have reported that vortioxetine-induced nausea decreases within 2 weeks of treatment initiation in evaluations conducted 3 months after starting therapy [24, 25]. In the present study, medical records confirmed symptom improvement in patients who experienced nausea and were followed up. However, approximately half of the patients who experienced nausea discontinued vortioxetine treatment. Because nausea was one of the most common adverse events observed in this study, strategies to manage gastrointestinal symptoms during the early treatment period may be important for supporting treatment continuation in young patients with depression. The findings of this study also suggest that concomitant gastrointestinal medication use was associated with a lower incidence of nausea. Mosapride, the most frequently prescribed drug in this study, is commonly used in clinical practice, and animal studies have reported its inhibitory effects on SSRI-induced vomiting [26]. SSRIs increase serotonin concentration, stimulate 5-HT3 receptors in the intestinal wall, and activate the vomiting center [13]. In contrast, mosapride promotes gastrointestinal motility in the stomach, duodenum, and colon via 5-HT3 receptor antagonism and 5-HT4 receptor agonism [26, 27]. Therefore, mosapride may have the potential to alleviate nausea in patients receiving vortioxetine.

Taken together, these findings suggest that concomitant gastrointestinal medication use may be associated with a lower incidence of nausea in patients treated with vortioxetine. However, this observation should be interpreted cautiously because of the retrospective study design and the potential influence of selection bias and confounding by indication.

This study has several limitations. The sample size was relatively small and the study was conducted at only two institutions, which limited statistical power and the ability to adequately control for potential confounding factors. Although age was adjusted for in the multivariable analysis, residual confounding cannot be excluded, particularly given the baseline age difference between the escitalopram and vortioxetine groups. In addition, the limited number of adverse-event outcomes may have resulted in unstable effect estimates. Standardized measures of depression severity, such as the Hamilton Depression Rating Scale (HAM-D), Montgomery–Åsberg Depression Rating Scale (MADRS), and Patient Health Questionnaire-9 (PHQ-9), were not routinely available in the medical records. Consequently, differences in baseline disease severity and their potential influence on antidepressant selection, treatment response, and adverse-event reporting could not be adequately evaluated. Furthermore, potential differences in disease severity between hospital and clinic patients could not be directly assessed. Patients treated in hospital settings may have had more severe or treatment-resistant depression than those treated in clinic settings, which may have influenced antidepressant selection, adverse-event reporting, and the observed outcomes. Another limitation relates to the assessment of adverse events. Adverse events were identified from physician-documented medical records and patient-reported symptoms recorded during routine clinical practice, and no standardized adverse-event assessment tool was used. Therefore, variations in documentation practices, symptom recognition, and reporting behavior may have affected the consistency of adverse-event ascertainment. In addition, important clinical factors such as gastrointestinal comorbidities, concomitant medications, and patterns of antidepressant use (initiation, maintenance, or switching) could not be fully evaluated.

Finally, because of the retrospective observational design, causal relationships cannot be established. In particular, the observed association between concomitant gastrointestinal medication use and a lower incidence of nausea should be interpreted cautiously, as selection bias and confounding by indication may have influenced the findings. Future prospective studies with larger sample sizes, standardized assessments, and more comprehensive clinical data are needed to further clarify the tolerability profile of vortioxetine and the potential role of gastrointestinal medications in the management of treatment-emergent nausea.

## Conclusions

In conclusion, vortioxetine was associated with a higher incidence of nausea than escitalopram among young patients with depression. Concomitant gastrointestinal medication use may be associated with a lower incidence of nausea; however, this observation requires confirmation in prospective studies. These findings provide additional real-world evidence regarding the tolerability of vortioxetine and may help inform the management of treatment-emergent nausea in young patients.

## Data Availability

The datasets generated and/or analyzed during the current study are not publicly available due to the protection of individual privacy but are available from the corresponding author on reasonable request.

## Abbreviations

SSRI: Selective serotonin reuptake inhibitor
S-RIM: Serotonin reuptake inhibitor and serotonin receptor modulator
ICD-10: International Classification of Diseases, 10th Revision
ORs: Odds ratios
CIs: 95% confidence intervals
